# *Helicobacter pylori cag* Pathogenicity Island's Role in B7-H1 Induction and Immune Evasion

**DOI:** 10.1371/journal.pone.0121841

**Published:** 2015-03-25

**Authors:** Taslima T. Lina, Shatha Alzahrani, Jennifer House, Yoshio Yamaoka, Arlene H. Sharpe, Bill A. Rampy, Irina V. Pinchuk, Victor E. Reyes

**Affiliations:** 1 Department of Microbiology and Immunology, University of Texas Medical Branch, Galveston, Texas, United States Of America; 2 Department of Pediatrics, University of Texas Medical Branch, Galveston, Texas, United States Of America; 3 Department of Medicine, Michael E. DeBakey Veterans Affairs Medical Center and Baylor College of Medicine, Houston, Texas, United States Of America; 4 Department of Pathology, Brigham and Women’s Hospital, Harvard Medical School, Boston, Massachusetts, United States Of America; 5 Department of Pathology, University of Texas Medical Branch, Galveston, Texas, United States Of America; 6 Department of Internal Medicine, University of Texas Medical Branch, Galveston, Texas, United States Of America; Jawaharlal Nehru University, INDIA

## Abstract

During *Helicobacter pylori* (*H*. *pylori*) infection CD4^+^ T cells in the gastric lamina propria are hyporesponsive and polarized by Th1/Th17 cell responses controlled by T_reg_ cells. We have previously shown that *H*. *pylori* upregulates B7-H1 expression on GEC, which, in turn, suppress T cell proliferation, effector function, and induce T_reg_ cells *in vitro*. In this study, we investigated the underlying mechanisms and the functional relevance of B7-H1 induction by *H*. *pylori* infection to chronic infection. Using *H*. *pylori* wild type (WT), *cag* pathogenicity island (*cag* PAI^-^) and *cagA* ^-^ isogenic mutant strains we demonstrated that *H*. *pylori* requires its type 4 secretion system (T4SS) as well as its effector protein CagA and peptidoglycan (PG) fragments for B7-H1 upregulation on GEC. Our study also showed that *H*. *pylori* uses the p38 MAPK pathway to upregulate B7-H1 expression in GEC. *In vivo *confirmation was obtained when infection of C57BL/6 mice with *H*. *pylori* PMSS1 strain, which has a functional T4SS delivery system, but not with *H*. *pylori* SS1 strain lacking a functional T4SS, led to a strong upregulation of B7-H1 expression in the gastric mucosa, increased bacterial load, induction of T_reg_ cells in the stomach, increased IL-10 in the serum. Interestingly, B7-H1^-/-^ mice showed less T_reg_ cells and reduced bacterial loads after infection. These studies demonstrate how *H*. *pylori* T4SS components activate the p38 MAPK pathway, upregulate B7-H1 expression by GEC, and cause T_reg_ cell induction; thus, contribute to establishing a persistent infection characteristic of *H*. *pylori*.

## Introduction

*Helicobacter pylori* (*H*. *pylori*) is a Gram-negative gastroduodenal pathogen. It infects >50% of the world’s population and is linked to chronic gastritis, peptic ulcer disease and gastric cancer (GC) [1–7]. *H*. *pylori* infection usually occurs in childhood and becomes established as a chronic infection. The persistent infection is a major risk factor in the development of GC, the second deadliest cancer worldwide. Overall, *H*. *pylori*-associated diseases result in considerable morbidity, mortality and societal costs.

Among the multiple virulence factors expressed by *H*. *pylori*, one that is noteworthy is encoded within a 40-kilobase chromosomal region known as the *cag* pathogenicity island (PAI), which is composed of more than 30 genes that encode for a type 4 secretion system (T4SS). Also, this island of genes includes the *cagA* gene that codes for the cytotoxin-associated gene A (CagA) protein which is the only known effector protein encoded in *cag* PAI and is a key virulence factor of *H*. *pylori*. Epidemiological studies showed that CagA^+^ *H*. *pylori* strains are associated with an increased risk of GC compared to strains of *H*. *pylori* lacking CagA [3,8,9]. The CagA protein is translocated into gastric epithelial cells (GECs) *via* the *H*. *pylori* T4SS [10,11] and once inside GECs the tyrosine residue at specific C-terminal Glu-Pro-Ile-Tyr-Ala (EPIYA) motif of CagA is phosphorylated [12,13]. The activated CagA interacts with several intracellular signaling mediators, mainly in the tyrosine phosphorylated mode [12,13], and activates some important signaling pathways to manipulate host immune regulation and deregulate GECs homeostasis for their survival [14,15]. In addition to CagA effector protein, T4SS also delivers *H*. *pylori* peptidoglycan (PG) cell wall fragments into host cells, which are recognized by the intracytoplasmic pattern-recognition receptor (PRR) nucleotide-binding oligomerization domain containing 1 (NOD1). The sensing of *H*. *pylori* PG by NOD1 activates NFκB and mitogen-activated protein kinases (MAPKs) and plays an important role in IL-8 production and pathogenesis [16–18].

Though the host mounts an immune response against *H*. *pylori*, it is inadequate to clear the infection. CD4^+^ Th cells are major effector cells in the immune responses to *H*. *pylori*. Although the numbers of CD4^+^ T cells in the gastric lamina propria with a memory phenotype increase during *H*. *pylori* infection, these T cells are hyporesponsive [19]. Because this hyporesponsiveness contributes to chronicity, there have been targeted efforts to understand the mechanisms employed by *H*. *pylori* to downregulate T cell responses. One mechanism involves the *H*. *pylori* vacuolating toxin A (VacA), which interferes with T cell function by downregulating IL-2 production, IL-2 receptor expression and T cell proliferation [20]. *H*. *pylori* also manipulate T cell function by eliciting regulatory T cells (T_reg_) which are frequently found in *H*. *pylori*-infected patients [21,22]. Because of their suppressive effect on T effector cells, T_reg_ cells further assist in the chronicity of infection. Although *H*. *pylori’*s T4SS importance in virulence is recognized because of its multiple effects on GECs, its role in modulating T cell function during *H*. *pylori* infection has not been well investigated.

Professional antigen presenting cells (APCs), such as dendritic cells and macrophages, are important in the regulation of the immune responses against *H*. *pylori* [23]. GECs are a major target for *H*. *pylori* infection and may function locally as APCs; however, their contribution to the response to *H*. *pylori* remains understudied. We have previously shown that GECs express cytokines and receptors that influence the T cell responses during *H*. *pylori* infection [24,25]. *H*. *pylori* can also use GECs as a fulcrum to inhibit T cell proliferation and cause T_reg_ cell induction from naïve T cells by inducing increased expression of the T cell co-inhibitory molecule B7-H1 on GEC [24,25]. B7-H1, also known as programmed death-1 ligand 1 (PD-L1), interacts with programmed death-1 (PD-1) receptor and causes downregulation of T cell activation. The mechanism that is used by *H*. *pylori* to increase B7-H1 molecule expression on GECs is unknown. In this study we investigated by using *in vitro* and *in vivo* systems the role of *H*. *pylori* T4SS and two mediators, CagA and PG, translocated into GECs in their increased expression of B7-H1. As both CagA and PG can activate several cell signaling pathways, we also investigated the cell signaling pathways involved in B7-H1 upregulation by *H*. *pylori*. Our results showed that *H*. *pylori* uses the p38 MAPK pathway to upregulate B7-H1 expression in GEC. Our data also highlighted the *in vivo* correlation of the presence of functional T4SS delivery system and B7-H1 upregulation with induction of T_reg_ cells in *H*. *pylori* infected mice.

## Materials and Methods

### Ethics Statement

All mice were kept under pathogen-free conditions, housed in polycarbonate cages on ventilated shelves, with food and water *ad libitum*. The experiments were performed according to an Institutional Animal Care and Use Committee (IACUC) approved protocol (# 0709046A). Feces from sentinel mice housed in the same room were routinely tested by PCR for murine pathogens, including pinworms, mouse parvovirus and consistently tested negative for each of these infections.

### Cell lines and bacterial cultures

Human GECs N87 and AGS were obtained from the American Type Culture Collection (ATCC) and the GEC line HGC-27 was obtained from RIKEN, The Institute of Physical and Chemical Research, Japan. These cell lines were maintained in RPMI 1640 with 10% fetal bovine serum (FBS) and 2 mM L-glutamine. Immortomouse stomach epithelium (ImSt) cells were derived from C57/Bl6 and were maintained in media as described by Whitehead et al. [26]. *H*. *pylori* strains 51B and 26695 as well as their corresponding isogenic *cagA* and *cag* PAI mutants were described previously [27,28]. *H*. *pylori* strains were grown on tryptic soy agar (TSA) plates supplemented with 5% sheep’s blood (Becton Dickinson, San Jose, CA) or on blood agar plates with 2.5 μg/ml of chloramphenicol (Technova, Hollister, CA) to maintain *cagA* ^-^ [28] and *cag* PAI^-^ strains at 37°C under microaerophilic conditions. *H*. *pylori* strain Sydney strain 1 (SS1) and PM-SS1 (pre-mouse SS1) [29] were used to infect mice. These strains were provided by Drs. J. Pappo (Astra) and Richard Peek (Vanderbilt Univ.), respectively.

### Animals

Female C57BL/6 mice were purchased from the Jackson Laboratory (Bar Harbor, ME). B7-H1^-/-^ mice [30] in C57BL/6 background were obtained from Dr. Arlene H. Sharpe (Harvard Medical School, Boston). Animals were tested negative for the intestinal *Helicobacter* spp. prior to their use in the experiments. Six-to-eight week old mice were used in the model of gastric *H*. *pylori* infection.

### Antibodies and cell signaling inhibitors

PECγ7-conjugated anti-human B7-H1 (clone M1H1), APC-conjugated anti-murine epithelial cell marker EpCAM (clone G8.8) and their isotype controls were purchased from eBioscience. Brilliant violet-conjugated B7-H1 (clone 10F.9G2) and the corresponding isotype control were purchased from Biolegend. The viability dye eFluor 780 (eBioscience, San Diego, CA, USA) was included in the experiments to control cell viability. For cell signaling inhibition the following inhibitors were used: CAY10512 (10 μM; Cayman chemical), AG-490 (100 ng/mL; Enzo Life Sciences, Farmingdale, NY), Wortmannin (100 nM; Calbiochem, Billerica, MA), and PD169316 (10 μM/mL; Cayman chemical, MI). PG-like molecule-NOD1 ligand-iEDAP (InvivoGen, San Diego, USA) was used to investigate the role of PG in B7-H1 expression.

### Infection of GEC with *H*. *pylori*

Before infecting the GEC with *H*. *pylori*, GEC were washed and their media replaced with antibiotic-free medium. The bacteria were resuspended in RPMI 1640 medium and used at a cell:bacteria ratio of 1:10.

### Flow cytometry

Flow cytometry was used for surface staining of B7-H1 on cultured GEC lines. Samples were collected after 24-h of incubation with the bacteria or after 12-h, 24-h and 48-h incubation to examine the kinetics of B7-H1 expression after iEDAP treatment. Prior to performing flow cytometry, cells were harvested, counted, their concentration/tube adjusted (10^6^ cells), washed and pre-incubated with normal mouse serum for 15 minute in ice. Cells were washed again and incubated with the corresponding conjugated antibodies or with isotype controls for 30 min in ice. After immunostaining the cells were washed twice with PBS and fixed with paraformaldehyde (1% in PBS). Cells were analyzed by flow cytometry on a LSRII instrument, where at least 10^4^ live events were analyzed on cultured human GEC and 10^5^ cells isolated from murine stomach in order to get 90% interval of confidence. The data were analyzed with BD FACSDiva software (BD Biosciences, San Jose, CA) and FlowJo (Tree Star, Inc, Ashland, OR).

### Real-Time RT-PCR

Real-time RT-PCR analysis was performed as previously described [28]. Briefly, RT real-time PCR was done according to the Applied Biosystems’s two-step RT real time PCR protocol (Applied Biosystems, Foster City, CA). The appropriate assays-on-demand^TM^ gene expression assay mix for human 18S and B7-H1 (a 20X mix of unlabeled PCR primers and TaqMan MGB probe, FAM^TM^ dye-labeled) and 2 μL of cDNA were added to the PCR reaction step. The reactions were carried out in a final volume of 20 μL using BioRad’s Q5 real-time PCR machine. The cycling parameters were as follows:2 min at 50°C, 10 min at 95°C (1 cycle) and 15 sec 95°C and one min at 60°C (40 cycles).

### NOD1 siRNA transfection

To knockdown NOD1 in GEC, the cells were transfected with siRNA for NOD1 by using the basic nucleofection kit for epithelial cells (Amaxa Biosystems, Gaithersburg, MD) according to the manufacturer’s instructions with a cocktail of 0.2 μM of siRNA or a negative control siRNA (Santa cruz Biotechnology, INC). For N87 cells, program T-005 was used. Knockdown of expression of NOD1 was verified by real time-RT-PCR.

### Murine infection and detection of B7-H1 expression, IL-10 production, FoxP3 expression, bacterial load and histopathology

C57BL/6 mice were orogastrically inoculated with 10^8^ CFU (in 100 μL of PBS/inoculation) of *H*. *pylori* SS1 or PMSS1 strains, three times within a week. Four weeks later sera were collected as the mice were euthanized and stomachs were removed. Stomachs were dissected longitudinally in 2–4 pieces. Stomach tissue was digested, GECs were isolated and used for immunostaining followed by flow cytometry analysis as we previously described [28].

Sera collected from *H*. *pylori* infected mice were examined for IL-10 using Luminex array (Millipore, Billerica, MA, USA) according to the manufacturer’s instructions. Samples were analyzed using Bio-Plex Manager software (Bio-Rad). After homogenization of mouse stomach tissue, mRNA was isolated and expression of IL-10 and FoxP3 were determined using RT-PCR.

Murine gastric tissue was homogenized and DNA was extracted using DNeasy Blood and Tissue Kit (Qiagen, Valencia, CA). After purification, the extracted DNA was used for the detection of *H*. *pylori* DNA by real time PCR using a protocol originally described by Rouessel et. al. [31]. RT-PCR for *H*. *pylori* 16S gene expression and bacterial quantification was performed as described previously [28]. For histopathology analysis one longitudinal strip of stomach was placed in 10% normal buffered formalin for 24-h at 4°C, transferred into 70% ethanol solution the next day, and stored at 4°C. Tissue was then embedded in paraffin and processed by H&E staining. The corresponding tissue sections were then evaluated and scored by a pathologist. Briefly, histopathologic grading was performed using visual analogue scales (mononuclear cells and neutrophils) derived from the Updated Sydney System classification of gastritis [32].

### Statistical analysis

Unless otherwise indicated, the results were expressed as the mean ± SE of data obtained from at least three independent experiments done with triplicate sets per each experiment. Differences between means were evaluated by analysis of variance (ANOVA) using student *t* test for multiple comparisons and considered significant if *p* was <0.05.

## Results

### *H*. *pylori* uses its T4SS to upregulate B7-H1 expression in GEC

**Fig 1:**
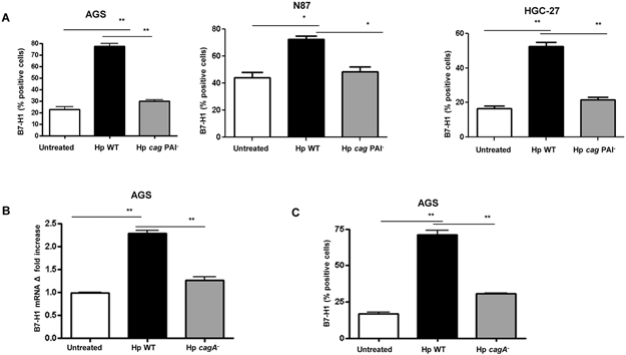
*H*. *pylori* uses T4SS to upregulate B7-H1 expression in GEC. (A) AGS, N87 and HGC-27 cells were infected with *H*. *pylori* 51B WT or 51B *cag* PAI^-^ strains at 10:1 *H*. *pylori*:GEC ratio for 24-h and B7-H1 expressed was measured by immunostaining followed by flow cytometry. (B) B7-H1 mRNA expression was analyzed using real-time quantitative RT-PCR in AGS cells. RNA was isolated from untreated and 2-h *H*. *pylori* 51B *cagA* ^+^ and *cagA* ^-^ infected GEC. mRNA level for B7-H1 was normalized to 18S rRNA and compared to the level of B7-H1 mRNA of untreated AGS cells (N = 9,* *P <*. *05)*. (C) Flow cytometry was done to measure B7-H1 expression on (C) AGS cells after 24-h infection with *H*. *pylori* 51B *cagA* ^+^ wild type (WT) and *cagA* ^*-*^ mutant strain. The data expressed as a percent positive cells. Isotype control value was subtracted from the data presented. N = 8,*P < 0.05, ** *P* < 0.01 and *** *P* < 0.001.

We previously showed using gastric biopsy samples from *H*. *pylori*-infected and uninfected subjects as well as different human GEC lines that *H*. *pylori* infection causes upregulation of B7-H1 expression by GEC. This increased expression of B7-H1 contributes to the inhibition of T cell proliferation and IL-2 production [24]. Interestingly, this increased B7-H1 expression by GECs was also noted to induce T_reg_ cells *in vitro* [25]. These responses are important contributors to the chronicity of *H*. *pylori* infection. Herein, we sought to determine the underlying mechanisms leading to B7-H1 upregulation by *H*. *pylori*. As the *H*. *pylori cag* PAI encoded T4SS is important in delivering bacterial products (i.e., CagA) that alter multiple properties of the gastric epithelium, we hypothesized that this virulence factor could influence B7-H1 upregulation. To test this hypothesis, we infected the human GEC lines (AGS, N87 and HGC-27 cells) with *H*. *pylori* 51B wild type (WT) strain which has an intact *cag* PAI and with an isogenic mutant strain lacking the *cag* PAI, *H*. *pylori* 51B *cag* PAI^-^. Our flow cytometry results showed that while the *H*. *pylori* WT strain caused a significant upregulation of B7-H1 expression in GECs, the mutant strain lacking *cag* PAI failed to upregulate B7-H1 expression (Fig 1A), which suggests a *cag* PAI-dependent upregulation of B7-H1 expression by *H*. *pylori*. Similar data were obtained with a different set of WT and *cag* PAI^-^ strains (*H*. *pylori* 26695). All experiments *in vitro* were performed with the three human GEC lines listed earlier to confirm that the results were consistent and not cell line-dependent.

### *H*. *pylori* T4SS translocated products CagA and PG both plays significant role in B7-H1 upregulation

**Fig 2:**
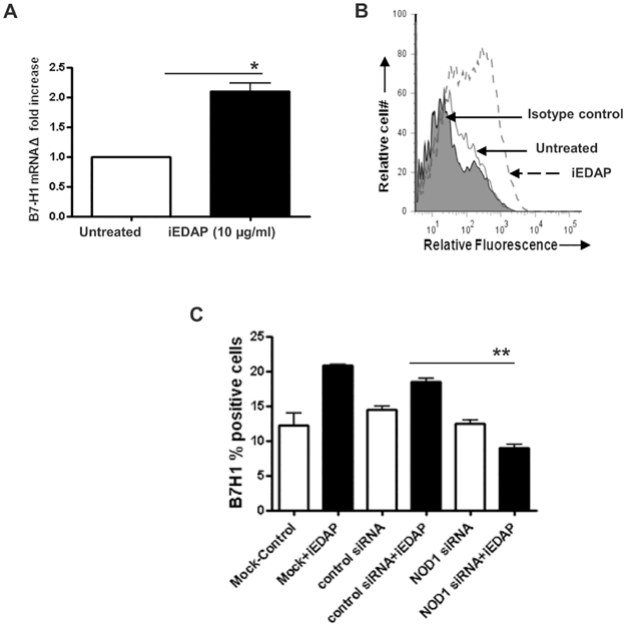
Recognition of PG by NOD1 causes induction of B7-H1 expression by GEC. (A) B7-H1 mRNA expression was analyzed using real-time quantitative RT-PCR in AGS cells. RNA was isolated from untreated and 2-h iEDAP (dipeptide present in PG) treated (10 μg/mL) cells. mRNA level for B7-H1 was normalized to 18S and compared to the level of B7-H1 mRNA of untreated AGS cells. N = 9, *P < 0.05. (B) Flow cytometry analysis of AGS cells stained for B7-H1 after exposure to 10 μg/mL iEDAP for 24-h showed increased expression in a representative histogram for AGS cells where the solid peak is the isotype control and (C) GECs were treated with NOD1 siRNA to knockdown NOD1 or with control siRNA and B7-H1 expression was analyzed by flow cytometry after iEDAP (10 μg/mL) stimulation. The means are shown as the results of duplicates in four experiments, n = 8,*P < 0.05.

*H*. *pylori* uses its T4SS to translocate into GECs the effector protein CagA and cell wall PG fragments [17,33], and each of these bacterial products has the ability to influence cell signaling pathways, pathogenesis and modulation of the physiology of GEC [17,34]. As our data showed, *H*. *pylori* mediated upregulation of B7-H1 depends on the presence of T4SS. To dissect the role of T4SS components in B7-H1 upregulation we investigated the role of T4SS translocated CagA and PG fragments on B7-H1 increased expression. To examine whether CagA plays a role in B7-H1 upregulation we infected human AGS cells with *H*. *pylori* 51B *cagA* ^+^ (WT) and a *cagA* ^-^ strain and assessed B7-H1 mRNA expression using real time RT-PCR. Fig 1B indicates more than a two-fold increase of B7-H1 mRNA expression from cells infected with *H*. *pylori* WT strain over the levels for untreated controls after 2-h of infection. In contrast, the *H*. *pylori cagA* ^-^ strain did not affect B7-H1 mRNA expression. Our flow cytometry data also showed that *H*. *pylori* 51B *cagA* ^+^ strains induced significant upregulation of B7-H1 expression on the surface of AGS cells compared to AGS infected with a *cagA* ^*-*^ mutant strain (Fig 1C). Similar data were obtained with the GEC lines N87 and HGC-27 and *H*. *pylori* 26695 WT and its isogenic *cagA* ^-^ mutant strains. These results further confirm that *H*. *pylori* bacteria use CagA protein for the upregulation of B7-H1 expression. As our data showed a partial dependence of *H*. *pyl*ori on CagA for B7-H1 upregulation, suggesting involvement of other components in this process, we also examined the role of PG in B7-H1 upregulation by GEC. GEC (AGS) stimulated with the synthetic PG analogue, iEDAP, that is a NOD1 ligand, had more than two fold upregulation of B7-H1 mRNA expression within two hours of incubation (Fig 2A). These results were confirmed by using an independent method to examine surface expression of the B7-H1 protein by flow cytometry. Our flow cytometry showed significant upregulation of B7-H1 expression on the surface of GECs after iEDAP stimulation (Fig 2B). We further confirmed our finding by inhibiting NOD1 expression by GECs by using siRNA nucleofection and examined B7-H1 expression after stimulating GECs with iEDAP. iEDAP stimulation failed to upregulate B7-H1 expression in GECs in which NOD1 expression was silenced (Fig 2C). Similar data were obtained with other GEC lines (N87 and HGC-27). Taken together, our data suggest that *H*. *pylori* uses T4SS delivered components CagA and PG to upregulate the T cell co-inhibitory molecule B7-H1 on GEC.

### Dose response and kinetics of PG mediated B7-H1 upregulation

**Fig 3:**
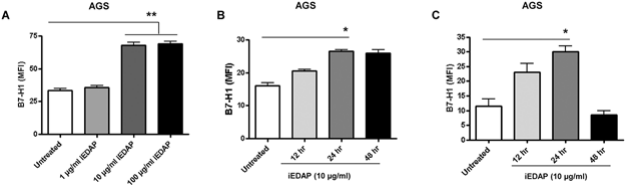
Kinetics and dose response of PG mediated B7-H1 upregulation. (A) Flow cytometry was done to measure B7-H1 expression on AGS cells after treating the cells with different concentrations (1 μg/ml, 10 μg/ml and 100 μg/ml) of iEDAP for 24-h. (B) AGS and (C) HGC-27 cells expressing B7-H1 at different time points after iEDAP treatment. B7-H1 expression was assayed by flow cytometry. The data expressed as mean fluorescence intensity (MFI). Isotype control value was subtracted from the presented data. The means ± SD are shown as the results of duplicates in four experiments, n = 8, * *P* < 0.05, ** *P* < 0.01 and *** *P* < 0.001.

To understand the involvement of PG in B7-H1 upregulation we examined the dose response and the kinetics of the response. To determine the dose response, GEC (AGS) were treated with ten-fold different concentrations of iEDAP and B7-H1 expression was analyzed by flow cytometry. The lowest concentration of iEDAP which elicited a significant upregulation of B7-H1 was 10 μg/ml, but it did not change much by increasing it to 100 μg/ml iEDAP as the response was similar to that with 10 μg/ml iEDAP (Fig 3A). Similar data were obtained with other GEC lines (N87 and HGC-27). Since 10 μg/ml iEDAP appeared to be optimal for the upregulation of B7-H1 in GEC, we used this concentration of iEDAP to examine the kinetics of the response. Both AGS and N87 (data not shown for N87 cells) cell lines, showed a progressive increase of B7-H1 expression detected as early as 12-h after stimulation with iEDAP and peaked at 24-h and stayed at the same level at 48-h (Fig 3B). However, HGC-27 cells showed a decrease at 48-h incubation (Fig 3C).

### B7-H1 upregulation in GEC involves the p38 MAPK pathway

**Fig 4:**
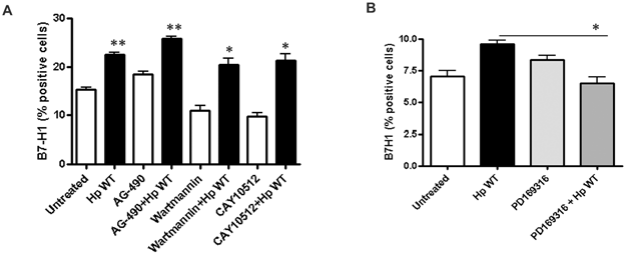
B7-H1 upregulation by *H*. *pylori* depends on p38 MAPK pathway. B7-H1 expression on N87 cells was measured by flow cytometry after treating the cells with (A) STAT3 inhibitor (AG-490), PI3K inhibitor (Wortmannin), NFκB inhibitor (CAY10512) and (B) p38 MAPK inhibitor (PD169316 10 μM/ml) for 1-h and infected with *H*. *pylori* for 24-h. The means ± SD are shown as the results of duplicates in four experiments, n = 8, * *P* < 0.05, ** *P* < 0.01 and *** *P* < 0.001.

CagA can activate several important cell signaling pathways including NFκB, MAPK, STAT3, and PI3K and cause proinflammatory cytokine production and modulation of GEC homeostasis [11,35–37]. PG fragments released by *H*. *pylori* and other gram negative bacteria are recognized by the intracellular NOD1 receptors and cause activation of NFκB and MAPK pathways [38]. Since our study showed a role for both CagA and PG in B7-H1 expression, we investigated the underlying cell signaling pathways used by these *H*. *pylori* mediators that could influence the modulation of B7-H1 expression. To that end, we used different pharmacological inhibitors directed against NFκB (CAY10512), p38 MAPK (PD169316), STAT3 (AG-490) and PI3K (wortmannin) pathways, which are known to be activated by *H*. *pylori* [12,35, 39–42]. Inhibition of NFκB, STAT3 and PI3K pathways had no effect on B7-H1 upregulation by *H*. *pylori* (Fig 4A); however, treating GECs with an inhibitor of the p38 MAPK pathway inhibited *H*. *pylori* mediated upregulation of B7-H1 expression (Fig 4B). These results suggest that *H*. *pylori* uses the p38 MAPK pathway to modulate B7-H1 expression in GEC.

### Upregulation of B7-H1 and induction of T_reg_ cells during murine *H*. *pylori* infection depends on T4SS

**Fig 5:**
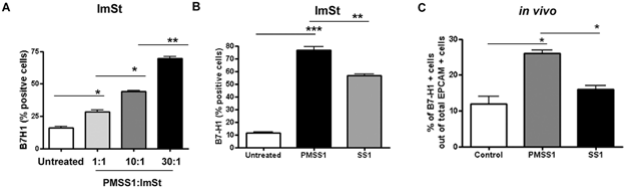
B7-H1 expression in mouse GEC and *in vivo* model depends on *H*. *pylori* T4SS. (A) ImSt cells infected with different ratio of *H*. *pylori* PMSS1:ImSt (1:1. 10:1, 30:1) for 24-h showed dose dependent upregulation of B7-H1 when analyzed by flow cytomery. (B) ImSt cells infected with PMSS1 strain (contains functional T4SS) showed higher expression of B7-H1 compared with cells infected with SS1 strain (lacks functional T4SS) as analyzed by flow cytometry. (C) C57BL/6 mice were challenged with *H*. *pylori* strain PMSS1, or with *H*. *pylori* SS1. Gastric mononuclear cells were isolated four weeks after *H*. *pylori* challenge using enzymatic digestion and level of the B7-H1 expressing epithelial cells (EpCam+) in the gastric mucosa from the cells was measured by flow cytometry.

In order to confirm the role of *H*. *pylori* T4SS in B7-H1 increased expression *in vivo*, we used the mouse model of infection in our study. Prior to using the *in vivo* model of infection, we sought to determine whether murine GECs express B7-H1 and whether their expression of B7-H1 is modulated by *H*. *pylori* infection. Thus, we used the ImSt murine gastric epithelial cell line and infected the cells with *H*. *pylori* PMSS1 strain, which is CagA^+^ and has functional T4SS. Using different *H*. *pylori* PMSS1: ImSt cell ratios (1:1, 10:1, 30:1) we showed that infection with this strain causes a significant increase of B7-H1 expression in murine GECs and the response is dose dependent (Fig 5A). Since our studies with human GEC showed involvement of CagA and PG in B7-H1 upregulation, we investigated the role of these components in the upregulation of B7-H1 by murine GECs by infecting ImSt cells with *H*. *pylori* PMSS1 strain, which showed significant upregulation of B7-H1 expression by flow cytometry, in parallel with ImSt cells infected with the *H*. *pylori* SS1 strain, whose T4SS is defective [43] and was found to be less effective at increasing of B7-H1 (Fig 5B). We also used these strains to infect human GEC (N87) and found significant induction of B7-H1 with PMSS1 infection but not with SS1 infection (See supplementary information for the data). These results were further validated *in vivo* since GECs isolated from *H*. *pylori* PMSS1-infected mice showed significant upregulation of B7-H1 expression after four weeks of infection compared to control mock-infected mice while GECs isolated from the *H*. *pylori* SS1-infected mice showed minimal increase in B7-H1 expression (Fig 5C).

**Fig 6:**
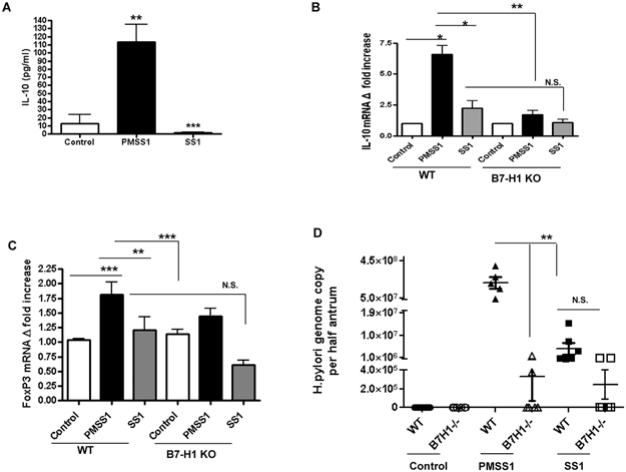
*H*. *pylori* uses its T4SS for T_reg_ cell induction and bacterial persistence. WT and B7-H1^-/-^ C57BL/6 mice were challenged with *H*. *pylori* strain PMSS1, or with *H*. *pylori* SS1. Mice were sacrificed after 4 weeks of infection. (A) Blood were collected and IL-10 was analyzed using luminex bead array. Data represents as mean ± SD (n = 12); *P <. *05*. Expression of (B) IL-10 and (C) FoxP3 mRNA in the mouse stomach was done by RT-PCR. (D) Infection rate was determined by quantification of *H*. *pylori* genome copy per half of antrum based on the analysis of *H*. *pylori* 16S gene amplification by real time PCR. Average bar of infection rate were calculated from five mice per group and demonstrated as a mean ± SD.

**Fig 7:**
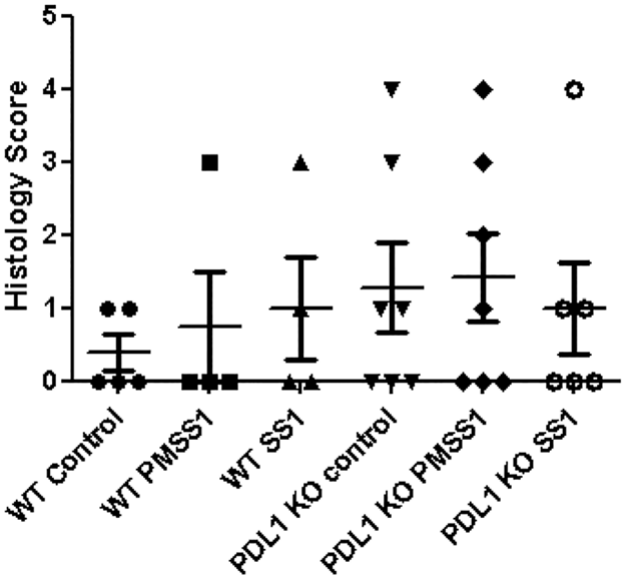
*H*. *pylori* T4SS-mediated B7-H1 and T_reg_ cell induction correlates with reduced gastric inflammation. WT and B7-H1^-/-^ C57BL/6 mice were infected with *H*. *pylori* strain PMSS1 or with *H*. *pylori* SS1. After 4 weeks of infection mice were sacrificed and histopathology was done in stomach tissue (H&E 20X) and evaluated by a pathologist blinded to the origin of the murine specimens.

To investigate the role of B7-H1 in the induction of T_reg_ cells *in vivo* we examined the T_reg_ cell population in WT and B7-H1^**-/-**^ mice infected with PMSS1 and SS1 strains after four weeks of infection. Serum cytokine analysis demonstrated that, in contrast to SS1-infected mice, PMSS1-infected mice have increased levels of IL-10, a cytokine that is associated with T_reg_ cell function (Fig 6A). The T_reg_ cell cytokine IL-10 and transcription factor FoxP3 mRNA expression were also higher in both WT and B7-H1^**-/-**^ mice infected with PMSS1 compared to SS1-infected mouse. However, PMSS1 infected B7-H1^**-/-**^ mice had lower expression of IL-10 and FoxP3 mRNA in their stomachs compared to the WT mice (Fig 6B and 6C). Analysis of bacterial loads in the stomachs showed increased bacterial loads in PMSS1 infected mice compared to SS1 infected mice and significantly reduced bacterial loads in the B7-H1^**-/-**^ mice compared to WT mice (Fig 6D). To investigate the functional relevance of the B7-H1 mediated increased T_reg_ cell response, gastric inflammatory response was analyzed after 4 weeks of infection. An early sign of chronic inflammation is the infiltration of mononuclear cells and eosinophils. A very scarce gastric infiltration of these cells could be observed in *H*. *pylori* PMSS1 infected mice compared to the SS1 infected mice (Fig 7). Interestingly, the B7-H1^**-/-**^ mice also showed increased presence of eosinophils compared to the WT mice. However, the observed differences did not reach statistically significant levels at the time that mice were examined. Taken together our *in vivo* data correlated with our *in vitro* data and showed that *H*. *pylori* T4SS and its ability to translocate CagA and PG play an important role in upregulating T cell co-inhibitory molecule B7-H1 on GEC, which in turn promotes induction of a T_reg_ cell type of anti-inflammatory response and aids in bacterial persistence.

## Discussion

*H*. *pylori* persistently colonizes the human stomach and elicits both humoral and cellular immune responses [44–46]. However, these immune responses do not clear the bacteria. CD4^+^CD25^hi^ FoxP3^+^ regulatory T cells (T_reg_) are present in higher numbers in the gastric mucosa of *H*. *pylori*-infected persons and play an important role in regulating the inflammatory response and inhibiting bacterial clearance [22,47]. Previously, we showed that *H*. *pylori* upregulates the expression of the T cell co-inhibitory molecule B7-H1 by human GEC, which in turn inhibit T cell proliferation [24] and cause induction of T_reg_ cells [25]. However, the mechanisms whereby *H*. *pylori* upregulate B7-H1 were unknown and could represent important targets in vaccine design. Using human GEC in an *in vitro* model complemented with an *in vivo* mouse model of infection which included both WT and B7-H1^-/-^ mice, we showed that *H*. *pylori* uses T4SS to translocate the effector protein CagA and PG cell wall fragments to upregulate B7-H1 expression by GECs. This increased expression of B7-H1 by GEC promotes the expansion of T_reg_ cells which may foster bacterial persistence. Our study also explored the underlying cell signaling pathways involved in this mechanism and showed that *H*. *pylori* uses the p38 MAPK pathway for the upregulation of B7-H1 expression.

B7-H1 (PD-L1) is a member of the B7 family which interacts with its putative receptor PD-1 and plays an important role in cell-mediated immune responses [48,49]. B7-H1/PD-1-mediated signaling plays a significant role in the regulation of T cell activation, tolerance, inhibition of T cell function and survival [50,51]. Our group initially demostrated that *H*. *pylori* upregulates B7-H1 in GEC [24]. Subsequent independent studies have confirmed that this pathway is targeted by various pathogens as it was noted that upregulation of B7-H1 occurs during gram-negative bacteria *Porphyromonous gingivalis* in oral squamous carcinoma cells and also hepatitis B virus-infected myeloid dendritic cells have been reported to increase B7-H1 [52,53]. Since *H*. *pylori* T4SS has been shown to play an important role in inflammation, pathogenesis and immune evasion mechanisms during *H*. *pylori* infection [34], we investigated its role in B7-H1 upregulation. First using *H*. *pylori* WT, *cag* PAI^-^ and *cagA* ^-^ mutant strains we showed that the upregulation of B7-H1 by *H*. *pylori* depends on the presence of T4SS and its effector protein CagA. Though in our initial studies we did not reveal a significant difference in B7-H1 expression levels in GEC infected with *H*. *pylori* WT and *cag* PAI mutant strain, this time using several GECs and infecting them with *H*. *pylori* clinical strain and its corresponding mutant we showed that *H*. *pylori cag* PAI and its effector protein CagA play an important role in upregulating B7-H1 at both the mRNA and protein levels. The previous attempt to determine the role of *H*. *pylori* T4SS in B7-H1 upregulation may not have revealed differences between wild type and *cagA* mutants in B7-H1 induction due to differences in the growth kinetics of the WT and mutant strains, which had not been studied. This time we studied the growth kinetics of the mutants and the corresponding parental strains and infected the GEC with the same CFU of the mutant and parental strains.

Our data also showed that *H*. *pylori*-mediated B7-H1 upregulation was only partially dependent on CagA injection. This suggested that other components of T4SS are also involved in this mechanism. Previous studies highlighted the importance of *H*. *pylori* T4SS secreted component PG in activation of MAPK and NFκB pathway and induction of inflammation [16]. Though recognition of PG by NOD1 is considered as important for host defense [17], the activation of these signaling pathways and production of IL-1β links them to the pathogenesis of several inflammatory diseases [37]. A recent study showed that *H*. *pylori* uses its HP310 protein for PG N-deacetylation which contribute to *H*. *pylori*’s survival in the host [54]. In this study we looked at the role of *H*. *pylori* PG in B7-H1 expression. Our flow cytometry and RT-PCR data clearly showed that *H*. *pylori* PG plays an important role in the upregulation of B7-H1 in GEC. By silencing NOD1 expression in GEC using siRNA nucleofection, we confirmed that recognition of PG by NOD1 contributes to this modulation of B7-H1 by *H*. *pylori*. To our knowledge, this is the first report showing the ability of *H*. *pylori* PG in modulating the immunoregulatory properties of GEC, specifically in contributing to inhibition of host T cell function.

As *H*. *pylori* mediated activation of host cell signaling pathways plays an important role in changing the homeostasis of GEC, which is very important for regulation of local T cell responses, we also determined the cell signaling pathways used by *H*. *pylori* to modulate B7-H1 expression in GEC. We focused on the cell signaling pathways that are known to be activated by *H*. *pylori* T4SS, e.g. NFκB, STAT3, MAPK and PI3K pathways [12,35,39–42]. Previous reports have highlighted the fact that *H*. *pylori* activates STAT3 to modulate host immune responses [14,36]. A recent study showed that CagA-dependent IL-8 mRNA induction also partially depends on STAT3. In that study, *H*. *pylori* CagA was reported to increase the bacterial lectin regenerating islet-derived (REG)3γ expression in GECs via activation of the STAT3 pathway, which allows *H*. *pylori* to manipulate host immunity to favor its own survival in the gastric environment [36]. MAP kinase activation is required for *H*. *pylori* IL-8 production [55]. MAPKs also regulate cell proliferation, differentiation, programmed death, stress, and inflammatory responses [56]. These observations suggest that through the activation of these pathways, *H*. *pylori* T4SS manipulates host immune regulation and deregulates gastric epithelial homeostasis for their survival. Using cell signaling inhibitors we showed that *H*. *pylori* uses p38 MAPK pathway to upregulate B7-H1 expression in GEC, since treating cells with the MAPK p38 inhibitor PD169316 prior to infecting the GEC with *H*. *pylori* inhibited B7-H1 induction by the pathogen. Thus our study showed another novel mechanism where *H*. *pylori* uses host cell signaling pathways to change the properties of GEC and thus makes a safer environment for bacterial survival.

The role of T4SS was further confirmed in a murine GEC line and *in vivo* using a mouse model of infection. Infection with the *H*. *pylori* PMSS1 strain, which contains a functional T4SS and can deliver CagA and PG into GEC, showed upregulation of B7-H1 molecule in both murine GEC line (ImSt) and also in the mouse model. However, *H*. *pylori* SS1 strain which lacks this delivery system failed to upregulate B7-H1 in both ImSt murine epithelial cells and in GEC isolated from infected murine gastric mucosa. Taken together our *in vitro* and *in vivo* data confirmed that *H*. *pylori* uses its T4SS component CagA and PG to increase T cell–coinhibitory molecule B7-H1 on GEC which plays an important role in controlling T cell activation.

*H*. *pylori* infected patients have increased numbers of T_reg_ cells (CD4^+^ CD25^high^ FoxP3^+^) in their gastric mucosa [47]. Though T_reg_ cells are important for suppressing overall T cell response during infection and cancer, they also inhibit protective effector T cell activity by producing anti-inflammatory cytokines IL-10 and TGF-β [57,58]. T_reg_ cells also inhibit memory T cell response in the periphery [22]. A previous study showed that vascular endothelial cells that can function as non-professional APC promoting the generation of T_reg_ cells from naïve CD4^+^ T cells in a B7-H1 dependent manner. T_regs_ cause inhibition of T cell proliferation and this process depends on B7-H1 [59]. Francisco LM et al., used B7-H1-/- APC to show that B7-H1 regulates T_reg_ cell development from naïve CD4^+^ T cells [60]. Previously by co-culturing *H*. *pylori*-infected GEC with naïve T cells our lab showed that *H*. *pylori*-mediated induction of B7-H1 co-related with induction of T_reg_ cell development from naïve T cells [25]. In the present study, we validated these findings *in vivo* and also determined the bacterial virulence factor involved in this mechanism. Our study showed that mice infected with *H*. *pylori* PMSS1 strain have increased levels of circulating IL-10, an important mediator of T_reg_ cell function and increased T_reg_ cell transcription factor FoxP3 mRNA expression in their stomachs after four weeks of infection. In contrast, infection with SS1 strain, which lacks a functional T4SS, failed to upregulate IL-10 production and FoxP3 expression. IL-10 produced by T_reg_ cells is critical for their function as FoxP3^+^ T cells lacking IL-10 are unable to suppress the development of gastritis and colitis [61]. In addition, by using B7-H1^-/-^ mice we showed that *H*. *pylori* uses B7-H1 to foster T_reg_ cell development since the B7-H1^-/-^ mice infected with *H*. *pylori* had reduced T_reg_ cell numbers compared to the WT mice. Although we observed a trend in the increase on inflammatory cells within the gastric mucosa of *H*. *pylori*-infected B7-H1^-/-^ mice compared to similarly infected wild type mice, it did not reach statistical significance at the time that we examined. Further investigation is needed to determine whether differences in inflammation are more pronounced at other time points. A previous study showed that T_reg_ cells play a protective role against gastric inflammation by reducing inflammation and ulceration in children [62]. In contrast, a study by Goll et al [63] suggested that IL-10 produced by T_reg_ cell contribute to the chronicity of *H*. *pylori* infection. Work by Rad *et al*. [64] showed that T_reg_ cells have dual function in inhibiting gastric inflammation and facilitating bacterial colonization. Though inhibition of bacterial-induced inflammation is important to reduce the extent of gastritis and ulcer formation, it is also important to clear the bacteria from the system to inhibit chronic infection. This study further supports our previous findings *in vitro* and showed that *H*. *pylori* mediated modulation of B7-H1 expression in GEC causes an increased T_reg_ type response and validate the hypothesis that T_reg_ induction aids the bacteria to survive in the gastric mucosa and further demonstrated that the pathogen is using its T4SS components for this mechanism. Taken together our studies showed for the first time how *H*. *pylori* use GEC as mediators to manipulate local T_reg_ cell response by using its T4SS components to aid in *H*. *pylori* persistence. These findings have significant importance as they provide insights into the *H*. *pylori* virulence factor involved in inhibiting host immune response and the underlying cell signaling pathway involved in this mechanism. Both the virulence factors and cell signaling pathways involved are potential targets to design a therapeutic or a vaccine to circumvent this highly prevalent human pathogen.

## Supporting Information

S1 Fig T4SS^+^ *H*. *pylori* PMSS1 strain upregulates B7-H1 expression in GEC. N87 cells were infected with PMSS1 strain (contains functional T4SS) or SS1 strain (lacks functional T4SS) at 10:1 *H*. *pylori*:GEC ratio for 24-h and B7-H1 expression was measured by immunostaining followed by flow cytometry. The data expressed as mean fluorescent intensity. Isotype control value was subtracted from the data presented. N = 8,*P < 0.05, ** *P* < 0.01 and *** *P* < 0.001.
(TIF)
