# Supplementary figures and images for: Helicobacter pylori cag Pathogenicity Island's Role in B7-H1 Induction and Immune Evasion
Source: PLoS One. 2015 Mar 25;10(3):e0121841. doi: 10.1371/journal.pone.0121841 (PMC4373751; doi:10.1371/journal.pone.0121841)

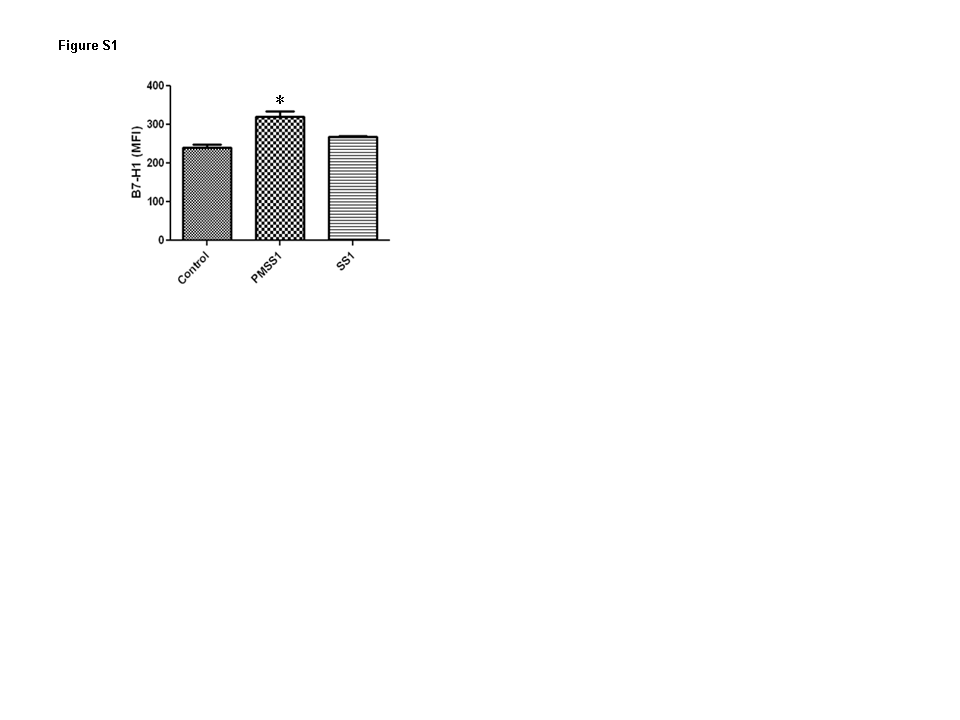

Supplement: S1 Fig — N87 cells were infected with PMSS1 strain (contains functional T4SS) or SS1 strain (lacks functional T4SS) at 10:1 H. pylori:GEC ratio for 24-h and B7-H1 expression was measured by immunostaining followed by flow cytometry. The data expressed as mean fluorescent intensity. Isotype control value was subtracted from the data presented. N = 8,*P < 0.05, ** P < 0.01 and *** P < 0.001. (TIF) [file pone.0121841.s001.tif]
